# Diabetes Care in French Guiana: The Gap Between National Guidelines and Reality

**DOI:** 10.3389/fendo.2021.789391

**Published:** 2021-11-30

**Authors:** Christine Sudre, Hélène Duplan, John Bukasakakamba, Mathieu Nacher, Pascale Peyre-Costa, Nadia Sabbah

**Affiliations:** ^1^ Regional Office of the Medical Service and Directorate of Risk Management Coordination of French Guiana, Cayenne, French Guiana; ^2^ Department of Endocrinology and Metabolic Diseases, Centre Hospitalier Andrée Rosemon, Cayenne, French Guiana; ^3^ Clinical Investigation Center, West Indies, French Guiana (INSERM CIC 14 24), Centre Hospitalier Andrée Rosemon, Cayenne, French Guiana

**Keywords:** diabetes, guidelines & recommendations, health inequalities, diabetes complication, French Guiana

## Abstract

**Introduction:**

French Guiana is a multicultural overseas territory in the Amazon, where precariousness and difficulties in access to care are widespread. The prevalence of diabetes is double that of other French departments, and cardiovascular morbidity and mortality is high. The objective of the study was to analyze the biological, clinical and therapeutic follow-up of patients with diabetes mellitus using exhaustive data and to correlate it with national and European recommendations.

**Material and Methods:**

Using the national health insurance data, 9079 and 10075 patients with diabetes mellitus were analyzed in 2018 and 2019, respectively. We analyzed antidiabetic treatments, medical, dental, and podiatric consultations, examinations prescribed as part of the annual follow-up, and home nursing care.

**Results:**

There was a significant increase over one year in the number of patients (+10%) with diabetes, mainly women (60%), and 31% were under 54 years of age, with a disparity depending on the area of the territory, the most isolated having less access to screening. Less than 56% of patients had HbA1c measurements twice a year, less than 43% had an annual renal check-up, only 19% had an ophthalmic check-up at least every two years, less than 25% had an annual dental check-up, and less than 4% had an annual follow-up with the podiatrist.

**Conclusions:**

Substandard diabetes monitoring is a major problem likely to increase morbidity and mortality. Adapting health care to the specificities of the territory is crucial, notably by formalizing the delegation of care to advanced practice nurse and non-healthcare professionals in precarious or geographically isolated areas.

## Introduction

**Figure 1:**
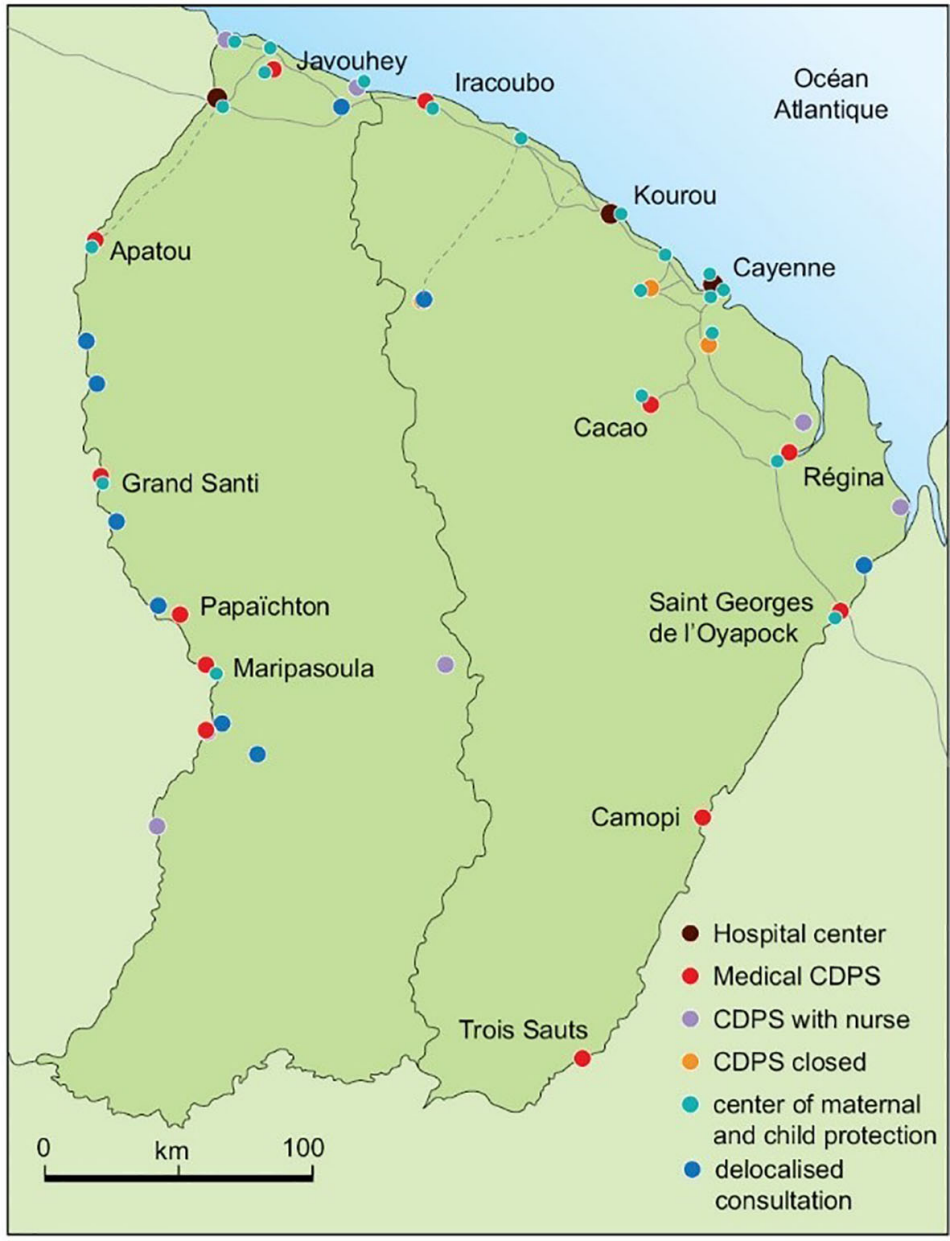
Map of French Guiana and the main hospitals and delocalized health centers. CDPS, community health care center.

French Guiana is one of the largest French overseas territories, with 95% of its 84,000 km2 covered by primary forest. With more than 22 ethnic groups, it is culturally diverse but this also raises difficulties in terms of therapeutic and especially educational management (1). French Guiana has 4 inter-communities which include 22 municipalities (detailed in Material and Methods). Most of the population is located on the coast, but some live in isolated areas and have great difficulty accessing the three main hospitals (Cayenne, Kourou and Saint Laurent du Maroni) (**Figure 1**). Indeed, the road infrastructure is insufficient, especially in the areas furthest from the major cities, and patients are sometimes forced to travel several days by canoe and by dirt road in order to access care. The medical and para-medical density is very low (8 times less specialists, 5 times less general practitioners than mainland France), and even more so in the areas located far from the coast (data from the Regional Medical Association). The prevalence of diabetes in French Guiana is 9.3%, as estimated by the 2014 health barometer, which is one of the highest in France, and women are more affected than men, unlike in other French territories (excluding the French West Indies) (2). The population of patients with treated diabetes has almost tripled in 12 years from 2467 in 2004 to 6795 patients in 2016 with a strong female predominance (61%). Moreover, the comparative mortality rate related to diabetes is 3 times higher than the national French rate per 100 000 inhabitants: 52 and 18% respectively (3), cardiovascular events being the main cause of death (the prevalence of stroke is one of the highest in France) (4). The renunciation of care –or non-demand of care— is important in French Guiana. The difficulties in accessing care have a multifactorial basis: language difficulties, cultural representations, precariousness, and the renunciation of care due to the remoteness of certain populations (1, 2, 5). Follow-up by specialists and adherence to treatments and dietary measures are important factors in avoiding diabetes complications (6, 7). Given the multiple contrasts in French Guiana, the management of diabetes in particular is very uneven across the territory.

The national guidelines for the follow-up of patients with pharmacologically treated diabetes recommend a minimum annual check-up. These recommendations have been integrated into the various guides of the French Health Insurance, such as the methodological guide for the remuneration of personal physicians based on public health objectives (8, 9).

Until now, the study of diabetes care in French Guiana rested mostly on samples from the main Hospital Cayenne which largely focusses on the Cayenne population basin. It was thus important to use more exhaustive data from various sites in French Guiana. Data on reimbursement of care provided to diabetic patients insured under the General Health Insurance Scheme, the Self-employed and Agricultural Professions Schemes, as well as data on hospitalizations and outpatient procedures and consultations (Medicalization of Information Systems Program) included in the national health data system make it possible to describe in detail the population treated for diabetes (insulin treated persons and no insulin treated persons) to assess the quality of prescription of antidiabetic treatments and care provided to these patients, particularly in terms of home nursing care and follow-up of biological and clinical examinations recommended to reduce diabetes-related complications.

The objective of the study is to evaluate the follow-up and prescription of treatments for diabetic patients in French Guiana, with details in different areas, and to establish their level of conformity with the national recommendations guide from data included in the national health system.

## Methods

### Selection of Treated Diabetic Patients

Oral or injectable anti-diabetic drugs (other than insulin) and insulin were selected from the Submission Identifier Code of the Drug Database, the reference database of allopathic drugs reimbursed by the French Health Insurance.

The data used to select the diabetics were taken from the inter-scheme consumption datamart. The persons included in the study were the beneficiaries of the General Health Insurance Scheme, the Social Scheme for Independent Professions and the Farmers’ Scheme of French Guiana who:

- received at least 3 deliveries (on different dates) of oral, injectable (other than insulin), or insulin antidiabetic drugs during the year, even when the treatments are delivered outside of French Guiana

- or at least 2 deliveries (on different dates) to the pharmacy of treatments for their diabetes during the year, even when treatments are delivered outside French Guiana.

Two populations were thus selected: patients with diabetes in 2018 and 2019. Those who died in 2018 or earlier were excluded from the 2018 population with diabetes and those who died in 2019 or earlier were excluded from the 2019 population with diabetes.

Assuming that insulin therapy is known a marker of severity in epidemiology, we distinguished between insulin-treated diabetes, which represents all persons with type 1 and partly with type 2 diabetes and non-insulin-treated diabetes, which essentially represents type 2.

The general characteristics of the diabetic population are: age and gender distribution of patients, analysis of drug strategies.

### Current French Recommendations for Patient With Diabetes Follow-Up

Description of the biological follow-up of patients: biological assays were identified from the following codes of the nomenclature of medical biology acts: HbA1c (1577), creatininemia with estimated glomerular flow (592, 593), microalbuminuria (1133), lipids (580, 590, 996 and 2001).

Description of patient clinical follow-up: retinographies and fundus examinations performed in private practice, in hospital or outpatient procedures over a period of 2 years were identified using the codes of the common classification of medical procedures of the French health insurance: BGQP007, BGQP140, BGQP002

Consultations in cardiology, ophthalmology, endocrinology, nephrology, dental consultations or podiatry, in private practice and in hospital outpatient procedures and consultations, were identified using the code corresponding to the medical specialty of the health professional performing them.

- At least two HbA1c (glycosylated hemoglobin) tests per year (the French National Authority for Health recommends at least two to four HbA1c tests per year). This assay can also predict the occurrence of microvascular complications of diabetes (8, 9).
- An annual urine sample test for microalbuminuria and an annual creatinine level test with an estimate of the glomerular filtration rate. This is used to search for the presence of nephropathy (10).
- At least one lipid test in a year, which is a significant associated cardiovascular risk factor (11).
- At least one consultation or fundus examination or retinography within 2 years to screen for retinopathy (12).
- An annual clinical foot examination by the treating physician or a podiatry consultation within a year, to prevent the risk of wounds and diabetic foot (13).
- A dental examination within a year, as periodontal disease is more important in diabetics (14).
- One examination by a cardiologist within one year (15).

### Nursing Follow-Up

The procedure codes used to trace the nursing follow-up of patients for diabetes are not specific. However, insulin-treated and/or non-insulin-treated patients were selected in their care pathway by the nursing act codes Nursing Technical Acts, Nursing Procedural Acts and Nursing Care Act; from the General Nomenclature of Professional Acts in France (16).

Thus, the procedure codes tracing nursing care in the general nomenclature of professional acts, Title XVI used for the study are as follows in supplementary file.

### Description of French Guiana Communities

It has 4 inter-communities which include 22 municipalities (detailed in Material and Methods): a community of agglomeration of the Centre Littoral (Cayenne, Matoury, Rémire-Montjoly, Montsinéry-Tonnégrande, Macouria and Roura), the community of Savanes (Kourou, Iracoubo, Sinnamary and Saint Elie), the community of West Guiana (Apatou, Awala-Yalimapo, Grand Santi, Mana, Maripasoula, Papaïchton, Saül and Saint Laurent du Maroni) and the community of East Guiana (Saint Georges, Ouanary, Camopi and Régina) (**Figure 1**).

### Ethics

Health insurance data in France are declared at the national level with a CNIL (The National Commission for Information Technology and Civil Liberties) authorization and authorization for data collection and analysis: CNIL notice of October 13, 2016, n°2016-316 and registration in the “National Health Data System “(with a registry for the French Guiana medical service department) n° DRSM-GUY-01/2021.

## Results

### Description of the Population

**Figure 2:**
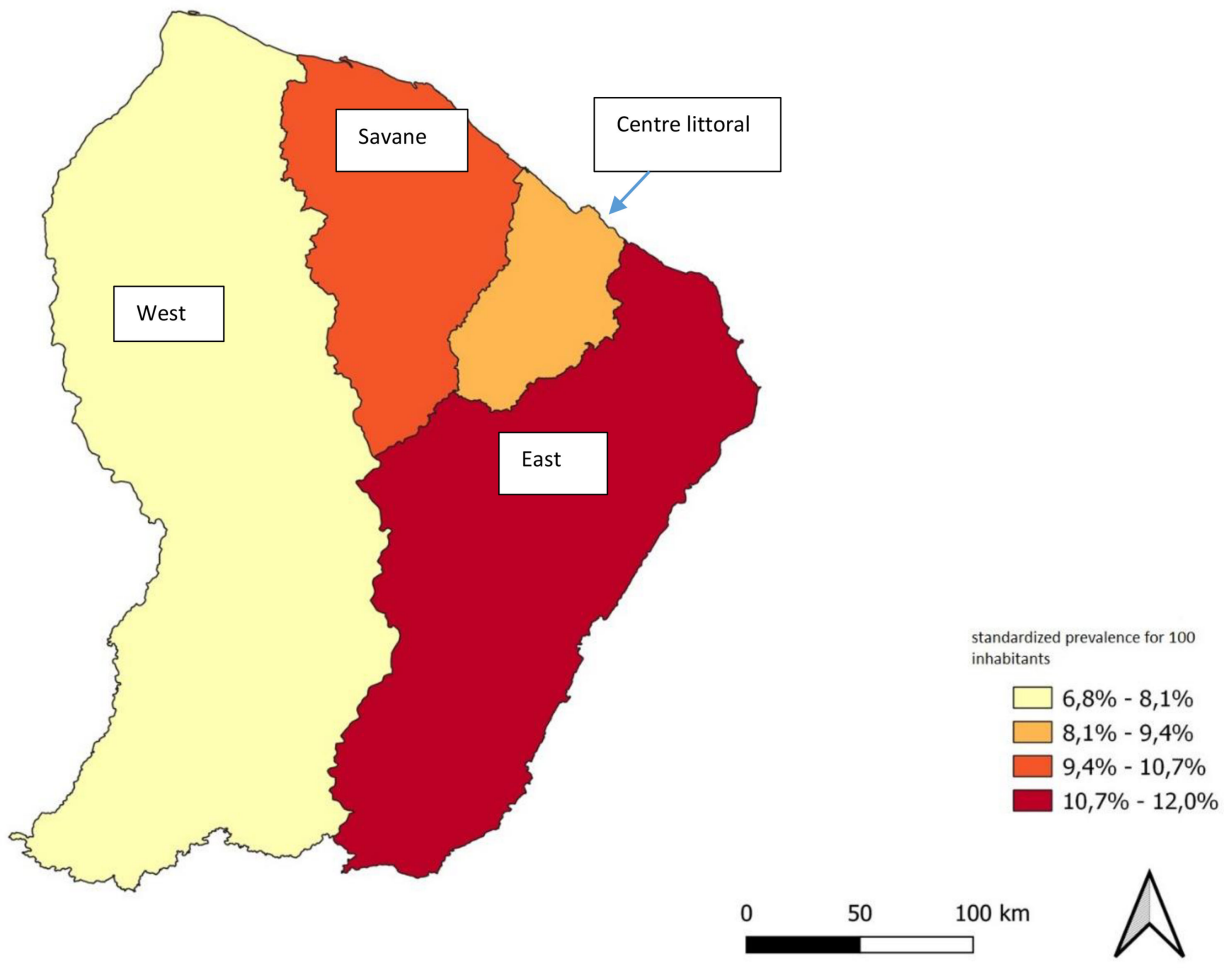
Map of the prevalence of diabetes in French Guiana according to the zones of the territory.

There were respectively 9079 and 10075 patients who were treated with antidiabetics or insulin in 2018 and 2019 (patients between 18 and 99 years). This does not include patients solely treated using hygienic and dietetic measures, nor patients without social rights (migrants who have been in the country for less than three months) or those who have state medical assistance (which is an emergency assistance for undocumented foreign patients without health insurance). We noted a 10% increase in the number of patients with diabetes between 2018 (9079 people) and 2019 (10075 people). The distribution of the number of diabetics was very inhomogeneous in the territory, with a standardized prevalence respectively for 2018 and 2019 of 12,0%, 9,7%, 8,9% et 6,8%, respectively in the territory of Eastern French Guiana, the Savane community, the central coastal area, and Western French Guiana (**Figure 2**).

**Figure 3:**
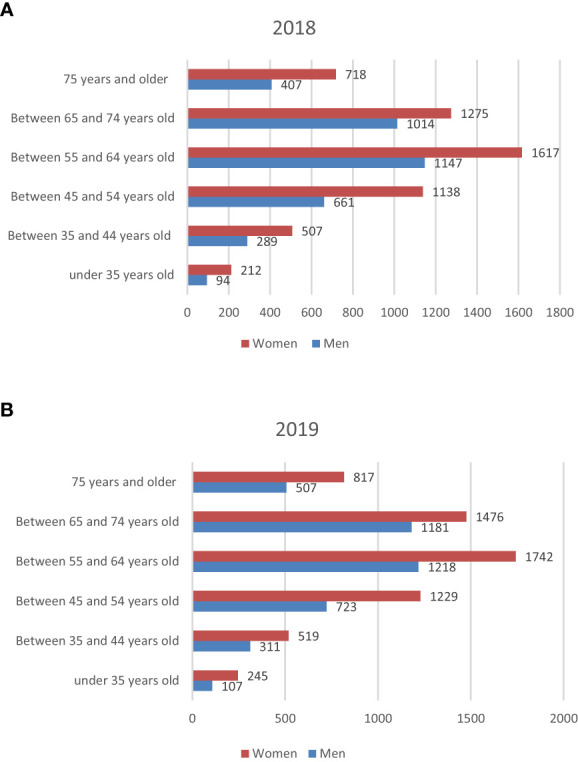
Number of patients with diabetes by age group and gender; **(A)** in 2018; **(B)** in 2019.

There was a predominance of women in all age groups with an average of 60% women and 40% men in 2019 (**Figure 3**).

The average age of diabetic patients was 60.3 years with at least 75% of the diabetic population being less than 69 years old. 32% of patients with diabetes were under 55 years of age (this includes type 1 diabetics), and 11% (over 75 years of age) (**Table 1**).

**Table 1:** Distribution of patients with diabetes by region in 2018 and 2019.

Communities	Number of inhabitants (as of 01/01/2017)	Diabetic population in 2018			Diabetic population in 2019		
		Total number of patients	Insulin-treated population N (%)	Non-insulin treated population N (%)	Total number of patients	Insulin-treated population N (%)	Non-insulin treated population N (%)
Urban community of Centre Littoral	138 920	5 848	2 138 (36,6%)	3 710 (63,4%)	6 456	2 418 (37,5%)	4 038 (62,5%)
Community of Savanes	30 645	1 211	402 (33,2%)	809 (66,8%)	1 311	410 (31,3%)	901 (68,7%)
Community of the West French Guiana	92 123	1 481	504 (34%)	977 (66,0%)	1 679	564 (33,6%)	1115 (66,4%)
Community of the East French Guiana	7 012	277	144 (52%)	133 (48%)	318	182 (57,2%)	136 (42,8%)
Outside French Guiana		262			311		

The distribution of non-insulin-treated and insulin-treated populations is detailed in **Table 1**. It was heterogeneous between different zones of the territory with patients in Western French Guiana treated with insulin more often (**Table 1**).

#### Biological and Clinical Monitoring

**Figure 4:**
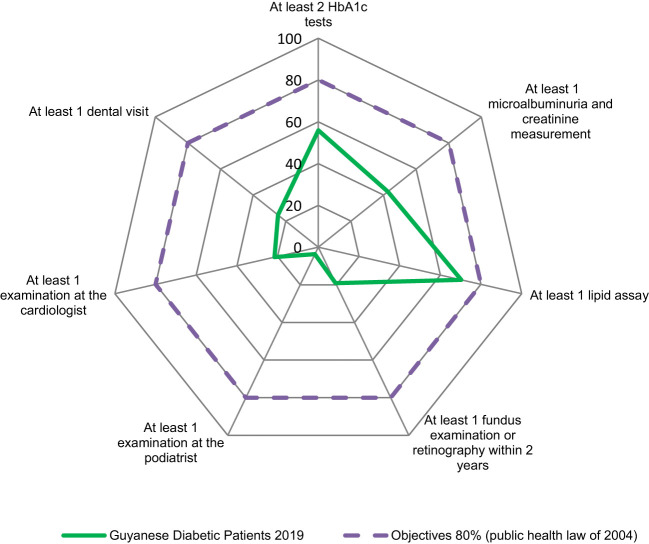
Adequacy of follow-up of patients with diabetes in French Guiana compared to French guidelines.

The proportion of patients who performed a lipid test at least once during the year was the only feature close to the 80% target (70.3%) (**Figure 4**).

Regarding recommended biological tests (**Figure 4**):

- HbA1c testing at least 2 times/year was found in 55.9% of treated diabetics in 2019, in similar proportions in insulin-treated and non-insulin-treated diabetics (56.8% and 55.3% in 2019); This proportion has increased slightly between 2018 and 2019 (55.3% vs 56.8% in insulin-treated diabetics and 54.5% vs 55.3% in non-insulin-treated diabetics).
- Fasting blood glucose testing was even less common in French Guiana’s treated diabetes population: 45.8% (n = 2937) of insulin-treated patients and 44% of non-insulin-treated patients (n = 1613) had at least 2 blood glucose tests in 2019. Of the total diabetic population, this represented 45.2% of patients (n = 4550). We also noted that 40.7% of patients, all treatments combined, did not have at least 2 blood glucose and HbA1c tests and that 17.6% did not have either of these two biological tests.
- The annual microalbuminuria and creatinine assay with glomerular flow estimation was performed in 42.6% of treated diabetics in 2019, with 4% more in insulin-treated patients (45.1% vs. 41.1%). The proportion of treated diabetics who performed each of the two assays at least once did not change between 2018 and 2019.
- Lipid testing at least 1 time per year was performed by 70.3% of treated diabetics in 2019, equally among insulin-treated and non-insulin-treated diabetics (69.5% vs. 70.8%). This proportion is equivalent to that observed in 2018 (70.9%).

**Figure 5:**
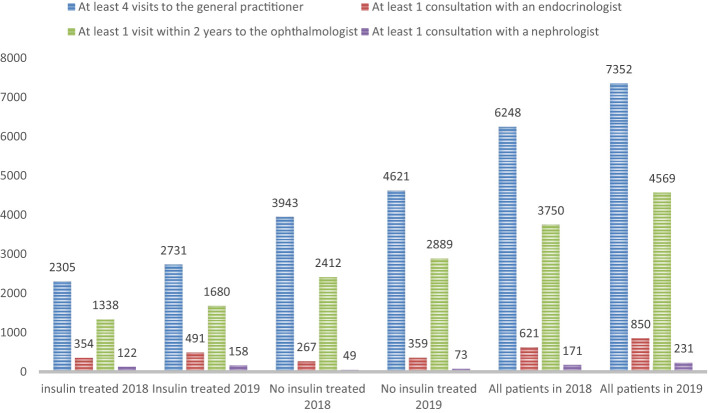
Distribution of medical visits recommended by the guidelines.

Regarding recommended clinical examinations (**Figures 4**, **5**):

- Ophthalmic fundus examination or retinography within 2 years was 14.5% in 2018 with a clear increase in 2019 (+4.6%), more marked in non-insulin treated patients (+5%) versus +3.8% in insulin treated patients.
- Annual cardiology follow-up was seen in 21.5% of patients with diabetes in 2019 (up 1.1% from 2018) and was more common in insulin-treated patients (23.6% versus 20.4% in 2019).
- Dental follow-up in 2019 was performed in ¼ of diabetic patients (24.7%) and was slightly more common in non-insulin treated diabetics (25.8% versus 22.9%). These proportions have not changed from 2018 (**Table 2**).
- In 2019, we found a podiatrist consultation in 3.7% of patients with treated diabetes with 2.5 times more among insulin-treated patients (6.2%) versus non-insulin-treated (2.4%). The proportion of insulin-treated diabetics performing a podiatrist visit increased by more than 1 point between 2018 and 2019. In contrast, no change was observed among non-insulin-treated diabetics (**Table 3**).

**Figure 4** summarizes the fraction of the population that complied with all the recommendations of the High Authority on Health with respect to the objective set by the 2004 Public Health Law (the national objective being that 80% of diabetic patients benefit from these recommended examinations).

**Table 2:** Distribution of dentist consultations according to the regions of the territory and according to the insulin therapy of patients with diabetes.

Communities	Population with diabetes treated in 2018				Population with diabetes treated in 2019				
	Total number of employees	Having had at least one visit to a dentist			Total number of employees	Having had at least one visit to a dentist			
		(N = 2229)				(N = 2493)			
		Total	Insulin treated	Non-insulin treated		Total	Insulin treated	Non-insulin treated
Urban community of Centre Littoral	5848	1546	501	1045	6456	1760	615	1145
		26,44%	32,41%	67,59%		27,26%	34,94%	65,06%
Community of Savanes	1211	307	104	203	1311	328	96	232
		25,35%	33,88%	66,12%		25,02%	29,27%	70,73%
Community of West French Guiana	1481	259	84	175	1679	270	73	197
		17,49%	32,43%	67,57%		16,08%	27,04%	72,96%
Community of the East French Guiana	277	45	27	18	318	54	34	20
		16%	60,00%	40,00%		16,98%	62,96%	37,04%
Outside French Guiana	262	72	NC	NC	311	81	NC	NC

**Table 3:** Distribution of podiatrist consultations according to the regions of the territory and according to the insulin therapy of patients with diabetes.

Communities	Population with diabetes treated in 2018				Population with diabetes treated in 2019			
	Total number of employees	Having had at least one consultation with a podiatrist			Total number of employees	Having had at least one consultation with a podiatrist		
		(N = 290)				(N = 379)		
		Total	Insulin treated	Non-insulin treated		Total	Insulin treated	Non-insulin treated
Urban community of Centre Littoral	5848	239	133	106	6456	325	195	130
		4,1%	55,6%	44,4%		5,0%	60,0%	40,0%
Community of Savanes	1211	33	14	19	1311	31	16	15
		2,7%	42,4%	57,6%		2,4%	51,6%	48,4%
Community of West French Guiana	1481	<10	<10	<10	1679	<10	<10	<10
Community of the East French Guiana	277	<10	<10	<10	318	<10	<10	<10
Outside French Guiana	262	16	NC	NC	311	22	NC	NC

#### Nursing Follow-Up

**Figure 6:**
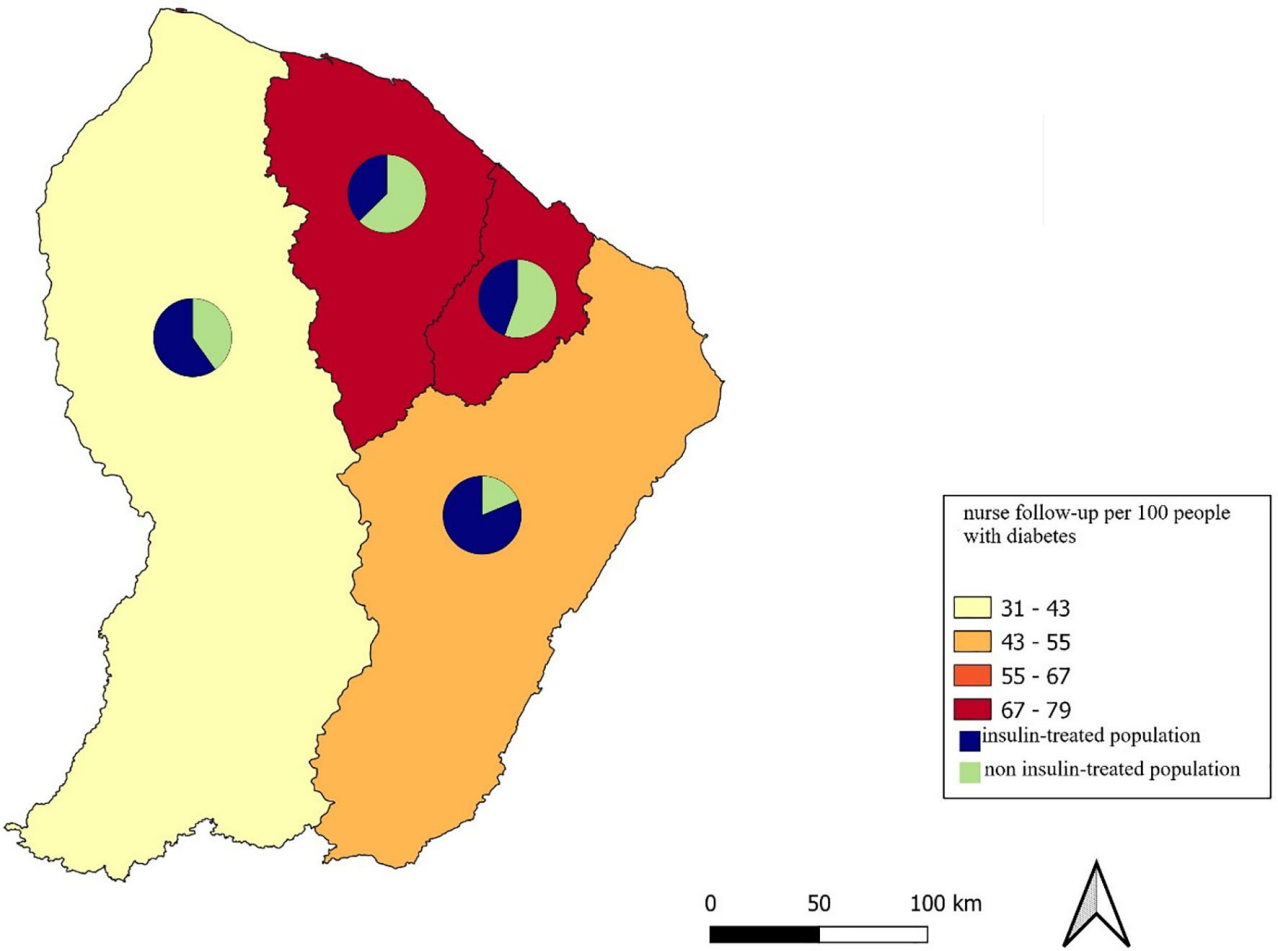
Distribution of nursing care in the territory.

In 2018, 72% of diabetic patients were followed by a home care nurse, compared with 64% in 2019 (**Tables 4A**, **B**). Regarding patients on antidiabetic drugs alone followed by a home care nurse, they were 55% in 2019 compared to 65% in 2018 (**Tables 4A**, **B**). There was a disparity in care according to the zones of the territory (**Table 4B**). Thus, the western zone, which is one of the most distant from the main hospital sites, has few nurses (**Figure 6**). The number of patients with diabetes is high in the eastern zone, particularly on the Brazilian border, but there is little nursing care in this zone.

**Table 4a:** Distribution of nursing procedures according to the nomenclature of health insurance procedures and according to the insulin therapy of patients with diabetes.

nursing act according to the French health insurance nomenclature		2018			2019	
	All patients with diabetes N (%) N = 9079	patients with diabetes treated with insulin N (%) N = 3265	patients with diabetes no treated with insulin N (%) N = 5814	All patients with diabetes N (%) N = 10075	patients with diabetes treated with insulin N (%) N = 3666	patients with diabetes no treated with insulin N (%) N = 6409
Nursing follow-up	6557 (72.2)	2749 (84,0)	3808 (65,5)	6479 (64,3)	2949 (80,5)	3530 (55,1)

**Table 4b:** Distribution of nursing follow-up according to the regions of the territory and insulin therapy.

Communities	Population with diabetes treated in 2018				Population with diabetes treated in 2019			
	Total number of patients	With nurse follow-up (N = 6557)			Total number of patients	With nurse follow-up (N = 6479)		
		Total	Insulin treated	Non-insulin treated		Total	Insulin treated	Non-insulin treated
Urban community of Centre Littoral	5848	4659 (79,7%)	1899 (40,8%)	2760 (59,2%)	6456	4548 (70,4%)	2028 (44,6%)	2520 (55,4%)
Community of Savanes	1211	1074 (88,7%)	374 (34,8%)	700 (65,2%)	1312	1040 (79,3%)	389 (37,4%)	651 (62,6%)
Community of West French Guiana	1481	511 (34,5%)	303 (59,3%)	208 (40,7%)	1679	558 (31,4%)	334 (59,9%)	224 (40,1%)
Community of the East French Guiana	277	142 (51%)	105 (73,9%)	37 (26,1%)	318	155 (48,7%)	126 (81,3%)	29 (18,7%)
Outside French Guiana		171	NC	NC		178	NC	NC

## Discussion

The present study, encompassing both private and public sectors, using the most thorough available data in French Guiana –the National system of health data—, highlights the very poor follow-up of our population. This focus had never been studied before, and here we used for the first time the health insurance system data to obtain a clear description of the situation. There was indeed a substantial gap between the recommended follow-up in the national (or American) guidelines and what is actually practiced in French Guiana (**Appendix 1**). Hence, although lipid anomalies were monitored among 70% of diabetic patients, only 21% of diabetic patients consulted a cardiologist at least once a year. Cardiovascular causes are the first cause of mortality in patients with diabetes (17). Cardiovascular complications are also important in French Guiana, particularly strokes, for which incidence is double that of other French territories (4).

A high proportion of patients with renal failure in French Guiana are diabetic, and notably 40% of those dialyzed for end-stage renal failure (18). Our data also shows that patients in French Guiana also did not comply well with the control of creatinine clearance and microalbuminuria, which are early markers of diabetic nephropathy (10). Monitoring of glycemic control was also very inadequate, with only 55.9% of patients with diabetes having HbA1c measurements at least twice a year. It is difficult to adapt treatments if patients do not measure their HbA1c regularly and are not educated to promote autonomy to achieve their glycemic target. Management at a very early stage of discovery of diabetes and achieving a target HbA1c (<6.5% at discovery) is an important point to avoid the complications of diabetes (19).

Only 14% of patients were able to perform a fundus or retinography every two years. The number of ophthalmologists/orthoptists performing retinographies is insufficient in French Guiana and almost all of them are in Cayenne and only one is in the hospital of Saint Laurent in western French Guiana. Early detection can reduce the prevalence and severity of retinopathy (20).

The number of podiatry consultations for the prevention of diabetic foot wounds was also very low. Prevention of diabetic wounds and feet care are essential to reduce the risk of amputation, and patients in precarious situations or in isolation are more at risk (21). In an American study, 74.9% of adults with diabetes had two or more HbA1c tests, 69.0% had a foot examination, 64.9% had an eye examination, 85.4% had a cholesterol test, but there were many disparities according to ethnicity, highlighting the role of culture, education and poverty in access to care (22). Depending on the culture and the level of knowledge, the patient’s interpretation of the risks linked to the absence of follow-up and the need to balance their diabetes are often very different, which requires adapting the educational messages according to the populations and the ethnic groups (23).

There is a great disparity in the distribution of patients with diabetes in French Guiana. The west has one of the lowest prevalence, probably due to less screening because it is an area where there is almost no road infrastructure, several days of dugout canoeing and hours of driving on tracks are the only means of accessing the hospital centers (1). However, it is of note that gastrointestinal nematodes are common in western French Guiana, and they have been shown to reduce the incidence of diabetes (24, 25). Both hypotheses for this distribution of diabetes however need to be formally tested.

In our study more than one third of the patients were treated with insulin (in Eastern French Guiana 50% received insulin), figures consistent with prior studies (2). Patients are prescribed insulin either because of the failure of other antidiabetic treatments or because of the presence of complications (26). The eastern sector borders on Brazil, particularly for the St Georges health center. It is very common for patients from northern Brazil to cross the border for urgent care and not return for follow-up. It is very likely that patients are screened late, when hyperglycemia is very important and following acute complications. While it is known that timely screening allows to decrease the use of insulin at 10 years, we are far from optimal conditions to do so (27). Between nearly two thirds (64% in 2019) and three quarters, (72% in 2018) of peoples with diabetes in our study were followed by a home health nurse; the increase in the number of patients with diabetes was not followed by an increase in the number of nurses, which may have explained the lower proportion in 2019. It is also possible that the opening of the territory’s first endocrinology-diabetes-nutrition specialty service in April 2017 has promoted patient autonomy and limited the use of insulin. However, the patients benefitting from home visits by nurses are numerous, and this reflects the frequent difficulties in terms of patient understanding and compliance in French Guiana. About one third of patients in the West were on insulin, and more than 80% of patients with insulin therapy had a home care nurse. Home care nurses provide insulin injections and are reimbursed by the health insurance system, but the density of nurses able to follow them at home is very low, and some places in the forest are inaccessible to caregivers. Thus, most often, even if insulin therapy is necessary, it is not instituted because the patients are not capable of managing the technical act of injection and dose adjustment on their own. The problem of educating the patient to reach autonomy, including in populations with difficulties in understanding and accepting treatment, is important to take into account in order to find management solutions adapted to each culture (1). In French Guiana, there are many patients with an immigrant background, and we note in the data from the health barometer study that they represent more than half of the patients with diabetes (2, 28). This multicultural character (more than 22 ethnic groups) is a source of wealth but also a difficulty for follow-up and therapeutic compliance (28–30).

Diabetes is a priority health problem in French Guiana and has a very high prevalence 9.3% in 2014 in the health barometer survey, with 38% of obesity and 39%overweight for people with diabetes (2). Persons with diabetes in French Guiana are more often in rural areas, more often precarious and are professionally less qualified, with more modest incomes than people without diabetes (2). The prevalence of women in our study population was high compared to other French departments; other studies have already highlighted this difference between the sexes (2, 31). The very frequent overweight and obesity in our territory (particularly in women) are one of the explanations (2, 31). French Guiana has changed a lot over the last 20 years with an increase in sedentary lifestyle. The present study highlights an increase of 10% in the number of diabetics between 2018 and 2019 even if it is difficult to extrapolate from only two years. However, the density of general practitioners is about half that observed in other departments in mainland France (42 vs 91 per 100,000 inhabitants). The gap is even greater for other specialties (24 vs 87 liberal specialists per 100,000 inhabitants), there is only one hospital endocrinologist and only one private practitioner in Cayenne (32). This poses a real public health problem in the context of major health inequalities and raises the reality of the lack of anticipation of care channels adapted to the growing incidence of metabolic pathologies.

One of the ways to improve these monitoring deficiencies and to improve the care of persons with diabetes in isolated and precarious areas would be to delegate tasks to non-medical staff, or advanced practice nurses in the vicinity in order to evaluate (systematic questioning, pulse examination, foot examination, portable retinography) the most at-risk patients requiring specialized consultations. Furthermore, it would be appropriate to develop delocalized biology in remote health centers to perform at least HbA1c and microalbuminuria, creatinine clearance.

The main limitation of our study, is a recruitment bias due to the lack of data from patients without treatment and those without social security. This means the number of diabetics is even higher than what we observed in this dataset—the largest analyzed hitherto in French Guiana. would probably have increased the figures found. Despite the above limitations, this is the first study in French Guiana to study such a large database of diabetic patients and the details of their routine care.

## Conclusions

Diabetes in French Guiana is a major public health problem, the prevalence of which is rapidly increasing and is twice as high as in other French territories. Our study shows that there was a huge gap between the follow-up of patients with diabetes in French Guiana and national recommendations. Precariousness, cultural diversity, the very low density of health professionals and geographical isolation are major elements explaining this deficient care. The development of proximity actions with health mediators (non-doctors), or advanced practice nurses, culturally adapted, in each sector of the territory, and trained to assist in the follow-up and compliance with treatment could be a solution.

## Data Availability Statement

The raw data supporting the conclusions of this article will be made available by the authors, without undue reservation.

## Ethics Statement

Ethics approval and consent. The project was conducted in accordance with the General Data Protection Regulations (GDPR) and in compliance with both French and European regulations (EU 2016 / 679), and reported to the National Institute of Health Data (INDS Health insurance data in France are declared at the national level with a CNIL (The National Commission for Information Technology and Civil Liberties) authorization and authorization for data collection and analysis: CNIL notice of October 13, 2016, n°2016-316 and registration in the “National Health Data System” (with a registry for the French Guiana medical service department) n° DRSM-GUY-01/2021.

## Author Contributions

Conceptualization: CS, HD, JB, PP, NS; Data curation: CS, HD, Formal analysis: HD, NS; Investigation: CS, HD, PP, NS Resources: CS, HD, PP, NS; Supervision: PP, NS; Validation: CS, HD, JB, MN, PP, NS Visualization: CS, HD, JB, PP, NS; Manuscript writing: CS, HD, JB, MN, NS. All authors contributed to the article andapproved the submitted version.

## Conflict of Interest

The authors declare that the research was conducted in the absence of any commercial or financial relationships that could be construed as a potential conflict of interest.

## Publisher’s Note

All claims expressed in this article are solely those of the authors and do not necessarily represent those of their affiliated organizations, or those of the publisher, the editors and the reviewers. Any product that may be evaluated in this article, or claim that may be made by its manufacturer, is not guaranteed or endorsed by the publisher.

## Abbreviations

HbA1c, glycosylated hemoglobin; DPPIV inhibitors, dipeptidyl peptidase inhibitors; GLP-1 analog, glucagon like peptid analog; SGLT2, sodium glucose co-transporter 2 inhibitors.
