# Supplementary material for: Diabetes Care in French Guiana: The Gap Between National Guidelines and Reality
Source: Front Endocrinol (Lausanne). 2021 Nov 30;12:789391. doi: 10.3389/fendo.2021.789391 (PMC8670498; doi:10.3389/fendo.2021.789391)
Supplement: Supplementary file 1 [file DataSheet_1.docx]

Supplementary file 1

- The nursing care procedure code, (DI) nursing care approach (Chapter ^1^, Article 11): Home care for a patient, regardless of age, in a situation of temporary or permanent dependence, excluding care under Article 12 of the same chapter. Designation of the nursing care procedures (DI)act: elaboration of the home nursing care approach necessary to carry out nursing care sessions or nursing clinical monitoring and prevention sessions of a dependent patient or to implement a personalized program in order to favor his maintenance, his insertion or his reintegration in the family and social framework.

- The code AIS: nursing acts of care, applicable to nursing sessions and home care of patients (Chapter ^1^, Article 11):

Care in the home for a patient, regardless of age, who is temporarily or permanently dependent**,** excluding care under section 12 of this chapter.

Designation of the act AIS 3: nursing care sessions, by half-hour session, at the rate of 4 maximum per 24 hours. The nursing care session includes all the care actions related to the functions of maintenance and continuity of life, aiming at protecting, maintaining, restoring or compensating the autonomy capacities of the person. These sessions cannot be prescribed for more than 3 months. Designation of procedure AIS 4: Weekly nursing clinical monitoring and prevention session, per half-hour session. This procedure includes, among other things, verification of compliance with treatment and its planning. The quotation of the clinical nursing monitoring and prevention sessions is subject to the prior elaboration of the nursing care approach. These sessions cannot be prescribed for more than 3 months.

**Home management of an insulin-treated patient**.

Procedure designation AMI 1: medical nursing procedures :

- Monitoring and observation of an insulin-treated diabetic patient whose condition requires regular adaptation of insulin doses according to the indications of the medical prescription and the result of the extemporaneous control, including the keeping of a monitoring sheet per session.

AMI Act 4 Designations:

- Weekly 30-minute clinical monitoring and prevention session for an insulin-treated patient over 75 years of age.

This quotation includes:

- Patient and/or family education;
- Verification of compliance with treatment and diet, screening for risk of hypoglycemia;
- Blood pressure control;
- Participation in the screening and follow-up of possible complications, in particular infectious, neurological, skin;
- The prevention of the appearance of these complications, in particular by maintaining proper foot hygiene;
- Keeping a monitoring sheet and transmitting information to the attending physician, who must be immediately alerted in case of risks of complications;
- The keeping, if necessary, of the liaison sheet and the transmission of useful information to the relatives or to the third person who substitutes for them.
- Heavy and complex dressing for an insulin-treated diabetic patient, requiring detersion with defibration.

In our study, we therefore analyze the nursing follow-up of diabetic patients with regard to the codes DI 1.5, DI 1, AIS 3, AIS 4, AMI 1 and AMI 4.

We compared the distribution of this nursing follow-up in insulin-treated and non-insulin-treated patients, according to age but also according to the distribution of nurses by community of communes.
