# Supplementary material for: Diabetes Care in French Guiana: The Gap Between National Guidelines and Reality
Source: Front Endocrinol (Lausanne). 2021 Nov 30;12:789391. doi: 10.3389/fendo.2021.789391 (PMC8670498; doi:10.3389/fendo.2021.789391)
Supplement: Supplementary file 2 [file DataSheet_2.docx]

| Guidelines | HBA1c | Microalbuminuria | Lipid assay | Fundus examination (or retinography) | Podiatrist examination | Cardiologist examination | Dental examination |
| --- | --- | --- | --- | --- | --- | --- | --- |
| French(1) | At least 1 /6 months | At least 1/ year | At least 1/year | At least 1/ 2 years | At least 1/ year | At least 1/ year | At least 1/ year |
| American (2) | At least 1/ 6 months | At least 1/ year | At least 1/ year | At least 1/ year | At least 1/ year | At least 1/ year | At least 1/ year |
|  |  |  |  |  |  |  |  |

| Reality in French Guiana | HBA1c | Microalbuminuria | Lipid assay | Fundus examination (or retinography) | Podiatrist examination | Cardiologist examination | Dental examination |
| --- | --- | --- | --- | --- | --- | --- | --- |
| Percentage of patients who have had their examination as recommended in 2019 (%) | 55.9 | 42.6 | 70.3 | 19.1 | 3.7 | 21.5 | 24.7 |

APPENDIX 1 French, European and American recommendations concerning the follow-up of patients with diabetes mellitus, and patients who have carried out their control as recommended by the national recommendations, in French Guiana

*ref*

1. Guide parcours de soins Diabète de type 2 de l’adulte [Internet]. Haute Autorité de Santé. [cité 14 juill 2021]. Disponible sur: https://www.has-sante.fr/jcms/c_1735060/fr/guide-parcours-de-soins-diabete-de-type-2-de-l-adulte

2. Association AD. Standards of Medical Care for Patients With Diabetes Mellitus. Diabetes Care. 1 janv 2003;26(suppl 1):s33‑50.
